# Wheat (*Triticum aestivum* L*.*) seedlings performance mainly affected by soil nitrate nitrogen under the stress of polyvinyl chloride microplastics

**DOI:** 10.1038/s41598-024-54838-8

**Published:** 2024-02-29

**Authors:** Ke Zhang, Mengge Wang, Yi Li, Xu Zhang, Kangqinglin Xiao, Chuang Ma, Xiaojing Zhang, Hongzhong Zhang, Yongle Chen

**Affiliations:** 1 School of Material and Chemical Engineering, Zhengzhou University of Light Industry, Zhengzhou, 430000 China; 2 Collaborative Innovation Center of Environmental Pollution Control and Ecological Restoration, Henan Province, Zhengzhou, 430000 China; 3 College of Earth and Environmental Sciences, Lanzhou University, No. 222 South Tianshui Road, Lanzhou, 730000 China

**Keywords:** Environmental impact, Plant stress responses

## Abstract

Microplastics are exotic pollutants and are increasingly detected in soil, but it remains poorly understood how microplastics impact soil and plant systematically. The present study was conducted to evaluate the effects of polyvinyl chloride microplastics (PVC-MPs) on wheat seedlings performance and soil properties. Under the stress of PVC-MPs, no new substance and functional groups were generated in soil by X-ray diffraction and the fourier transform infrared spectroscopy analyses, whereas the diffraction and characteristic peaks and of soil was affected by PVC-MPs. Wheat seedlings shoot biomass and soil nitrate nitrogen were significantly inhibited by PVC-MPs. Chlorophylls were not significant affected by PVC-MPs. Superoxide dismutase, catalase, and peroxidase activities in wheat seedlings increased, while malondialdehyde and proline contents decreased significantly. Redundancy analysis displayed that wheat seedlings traits can be largely explained by soil nitrate nitrogen. Our results indicate that PVC-MPs have more significant influence on soil structure than on soil substance composition. Moreover, even though antioxidant enzyme activities were improved to respond the stress of PVC-MPs, wheat seedlings are not severely impacted by PVC-MPs. Besides, soil nitrate nitrogen is the main factor on wheat seedlings performance and wheat seedlings are prone to ensure the root growth under the stress of PVC-MPs.

## Introduction

Microplastics are derived from the plastics that are widely used in various industries, due to their low cost, durability, and malleability^1,2^. In recent decades, microplastics have aroused wide concern, and terrestrial ecosystems are believed to suffer more microplastics input than aquatic systems^3,4^. In terrestrial ecosystems, agricultural soil is thought to be the most important reservoir of microplastics through greenhouse materials, soil conditioners, mulch films, biosolids, wastewater, and atmospheric fallout^5^. The concentration of microplastics in the largest vegetable production soil ranges from 310 to 5698 items kg^−1^^6^. In the context of globalization, microplastics in soil are likely to increase continuously and cause damage to soil biota, and even accumulate in the food chain^5,7^. Hence, understanding the effects of microplastics on soil–plant system is of great necessity to evaluate the ecological risk of microplastics.

Due to the low oxygen and light conditions for degradation, microplastics may retain in soil for a hundred years, which will lead to a series of changes in soil environment^8^. For example, polyester fiber (0.4%, w/w) can significantly reduce soil bulk density and increase water holding capacity and evapotranspiration, while polyethylene fragments and polyamide (2.0%, w/w) have less effect on soil hydraulic properties^9^. A high level of polypropylene microplastics (28%) can promote fluorescein diacetate hydrolase (FDAse), leading to the enhancement of the hydrolysis activity on organic matter by microorganisms, and thereby reducing the decomposition rate of soluble organic matter and significantly increasing soil nutrients^10^. However, de Souza Machado et al.^7,9^ showed that changes in soil FDAse activity depend on the types of microplastics. Moreover, even for the same type of microplastics, distinct different results can be found. The high concentration of polyvinyl chloride microplastics (PVC-MPs) inhibited the abundance of soil *Acidobacteria* in acid cropped soil, while it can promote soil *Acidobacteria* abundance in neutral cropped soil^8,11^. Hence, the effects of microplastics on soil environment are still unclear due to the variations of types and sizes of microplastics.

It has been confirmed that microplastics can indirectly affect plant growth by altering the soil environment^12,13^. For example, Wang et al.^14^ found that 10% (w/w) polylactic acid microplastics can significantly reduce root growth by affecting rhizosphere microbial arbuscular mycorrhizal fungi. Microplastics can also directly affect plant growth by blocking plant roots and entering plant organs^3,4,15^. The performance of plant also can be influenced by the disturbed photosynthetic rate and antioxidant enzyme activities by microplastics^16,17^. Generally, microplastics can affect plant performance in a variety of ways, but the effects of microplastics on plant also depend on microplastics type and content^11,13^. Hence, it is necessary to explore the effects of different types, sizes, and contents of microplastics on plants. It is therefore of supreme importance to conduct further research of microplastics on soil–plant system to fully understand the risk effects of microplastics.

In present study, a field experiment was designed to explore the effects of PVC-MPs, one of the dominant plastics in agricultural soil, on soil and wheat (*Triticum aestivum* L.) system. This study aimed to (1) display the dynamic of soil microbial biomass, enzymes, and nutrients triggered by PVC-MPs; (2) exhibit the alteration of wheat seedlings growth and physiological traits triggered by PVC-MPs; and (3) reveal the restricting factors on wheat performance under the stress of PVC-MPs. This study will be conducive to further improving our understanding of the risk of microplastics in agroecosystems.

## Materials and methods

### Materials preparation

The tested PVC-MPs with a diameter of 15 μm was purchased from Jintian Plastic Raw Material Co, Ltd., China. Morphology of PVC-MPs was characterized by scanning electron microscopy (SEM) (JSM-6490LV, Japan) (Supplementary Fig. S1). The tested wheat (*Triticum aestivum* L., Zhengmai No. 9023) seeds were purchased from the Henan Academy of Agricultural Sciences, China. The volume of pot used for the experiment was about 2.5 L with the height, top diameter, and bottom diameter of 11 cm, 18 cm, and 16 cm, respectively.

The experimental soil was sampled from the northwest suburb of Zhengzhou, China. The sampling farmland was not covered with plastic film, and it was a long-term rotation of wheat, corn, and soybean, with no other pollution. In the farmland, four corners and centers of the 5 m × 5 m area were selected. The soil in the upper of 0–20 cm was collected. After animal and plant residues, stones, and other debris were removed, soil was air-drying naturally and passed through the 2 mm sieve for experiment. The basic characteristics of the soil was shown in Supplement Table S1.

### Experiment design and sample collection

PVC-MPs contents in present study were designed as 0%, 0.1%, 1%, and 10% (w/w), respectively. According to the reported effects of MPs on plant–soil system and the slow degradation rate and the increasing use of agricultural plastics, the detected MPs in soil is expected to increase^7,14^. The highest dose of PVC-MPs far beyond other research was designed to evaluate its effect on soil and plant system in the long term^18^. The mixtures of soils and PVC-MPs were put into pots. A total of 12 pots were set for the experiment with each treatment was repeated three times. Based on the principle of completely random design, these pots were randomly imbedded into the sampling farmland soil and the surface of pots was on the same level of farmland soil surface.

Before sowing, wheat seeds with intact, uniform grain size, and full grain were selected and disinfected with 1% NaClO solution for 20 min, then rinsed with distilled water several times, and finally soaked in warm water for 12 h. Each pot evenly sowed 15 wheat seeds with depth is about 2 cm. After sowing, soil moisture was kept at about 60% of field capacity by soil moisture monitor (LD-WS, China) and the growth of wheat seedlings was observed every day. After 30 days, the wheat seedlings were sampled for determining leaves enzymes and biomass. Meanwhile, soils in each pot were collected and divided into two parts. One part was stored in the refrigerator at 4 °C for the determination of soil microbial biomass, soil enzyme activities, soil nitrate nitrogen (NO_3_^−^–N), soil ammonium nitrogen (NH_4_^+^–N), and soil available phosphorus (AP). Another part was used to measure soil water content, soil organic carbon (SOC), and soil total phosphorus (TP).

### Measurements of wheat and soil parameters

The height of wheat seedlings was determined by a vernier caliper. Wheat seedlings were divided into shoot part and root part and dried in a 60 ℃ incubator to a constant weight. After that, the dry weight of seedlings was determined for calculating root biomass (R), shoot biomass (S), and its ratio (R/S). The contents of chlorophyll *a*, chlorophyll* b*, and carotenoid were measured and calculated based on the equations in Wang et al.^19^. The contents of superoxide dismutase (SOD), catalase (CAT), guaiacol peroxidase (POD) and glutathione (GSH) in wheat shoot were determined by enzyme kit (Shanghai Enzyme Link Biotechnology Co., China). Malondialdehyde (MDA), proline, and soluble protein contents were measured by thiobarbituric acid method, sulfosalicylic acid method, and coomassie brilliant blue method, respectively^20^.

Soil microbial biomass carbon (MBC) and microbial biomass nitrogen (MBN) were measured by Elementar Vario TOC/TN analyzer (Germany). Soil microbial biomass phosphorus (MBP) was measured by inorganic phosphorus method. Soil catalase, urease, alkaline phosphatase, sucrase, and protease activities were determined by potassium permanganate titration, sodium phenol-sodium hypochlorite colorimetry, sodium phenyl phosphate colorimetry, 3,5-dinitrosalicylic acid colorimetry, and ninhydrin colorimetry methods, respectively. Soil organic carbon (SOC) was measured by potassium dichromate volumetric method. Soil nitrate nitrogen (NO_3_^−^–N) and ammonium nitrogen (NH_4_^+^–N) were determined by UV spectrophotometry after leaching with potassium chloride. Soil available phosphorus (AP) was determined by 0.5 mol/L NaHCO_3_ method. Total phosphorus (TP) in soil was determined by sodium hydroxide melting-molybdenum antimony colorimetric method. These determination methods of soil chemistry properties were derived from the book of Soil and Agricultural Chemistry Analysis ^21^. The mineralogy of PVC-MPs and soil samples were measured by X-ray diffraction (XRD) (D8 Advance, Germany). The fourier transform infrared spectroscopy (FTIR) was used to identify the variation functional groups of soils after the addition of PVC-MPs (Bruker Tensor27, Germany).

### Statistical analysis

One-way ANOVA was used to give a statistically significant result of wheat growth physiological traits and soil properties, and Student-Newman Keuls was run to display the differences of wheat growth physiological traits and soil properties among treatments. Redundancy analysis (RDA) was used to study the effects of soil properties on wheat traits. SPSS 18 and Origin 8.5 (Origin Lab, USA) were used for data analysis and graphing. Data are expressed as mean ± standard error.

## Results

**Figure 1:**
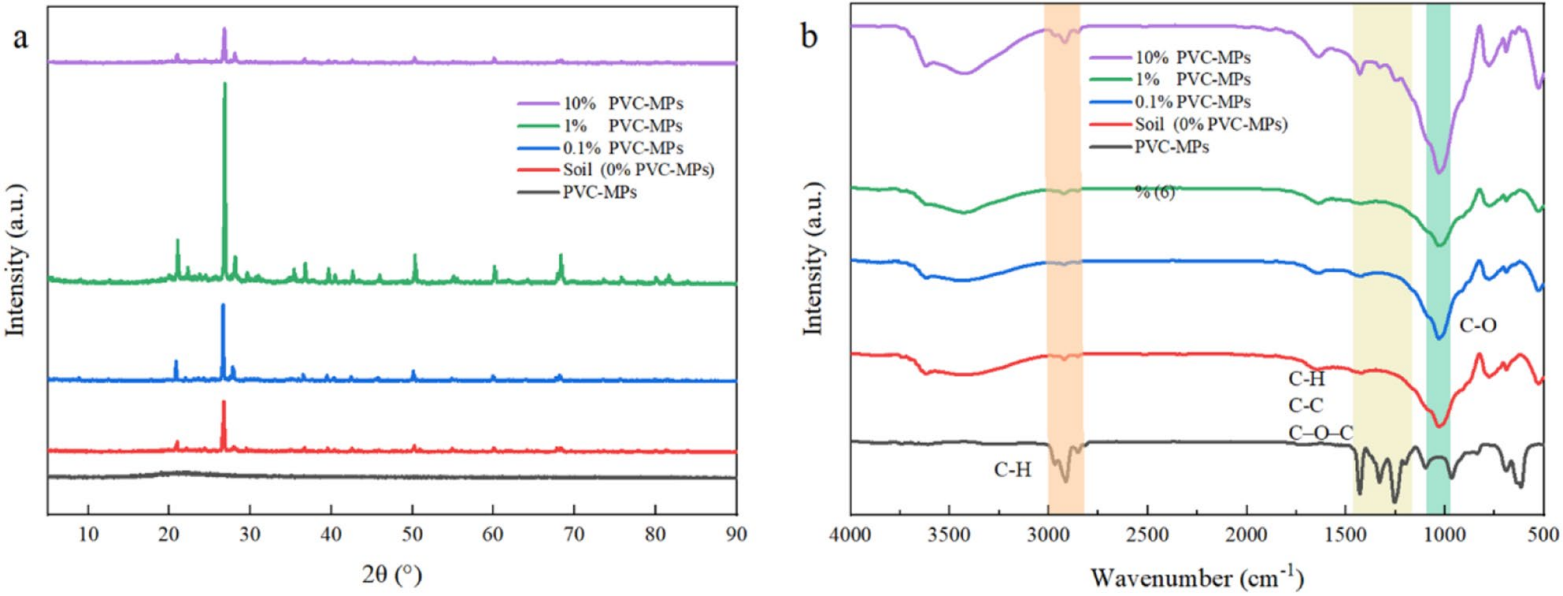
X-ray diffraction and fourier transform infrared spectroscopy analyses on PVC-MPs, soil, and treatments. One-Way ANOVA was used to analyze effects of PVC-MPs on soil properties and wheat growth and physiological parameters. They were presented in histogram. In each histogram, the different lowercases represent the significant difference at *P* < 0.05 level.

The variations of crystalline and functional groups of PVC-MPs, soil, and soil with PVC-MPs were carried out by XRD and FTIR (Fig. 1a and b). As shown in Fig. 1a, PVC-MPs did not show apparent diffraction peak. As for the soil (control) and soil with PVC-MPs, the significant sharper of diffraction peak occurred at 27° (Fig. 1a). PVC-MPs have special absorption peaks in the ranges of 2800–3000 cm^−1^ and 1120–1475 cm^−1^ (Fig. 1b). The soil had special absorption peaks at 2800–3000 cm^−1^ and 1020–1080 cm^−1^, representing the functional groups C–H and C–O, respectively (Fig. 1b). The addition of PVC-MPs did not change the soil functional groups, while the functional groups of C–H and C–O in soil were enhanced by 10% PVC-MPs (Fig. 1b).

**Figure 2:**
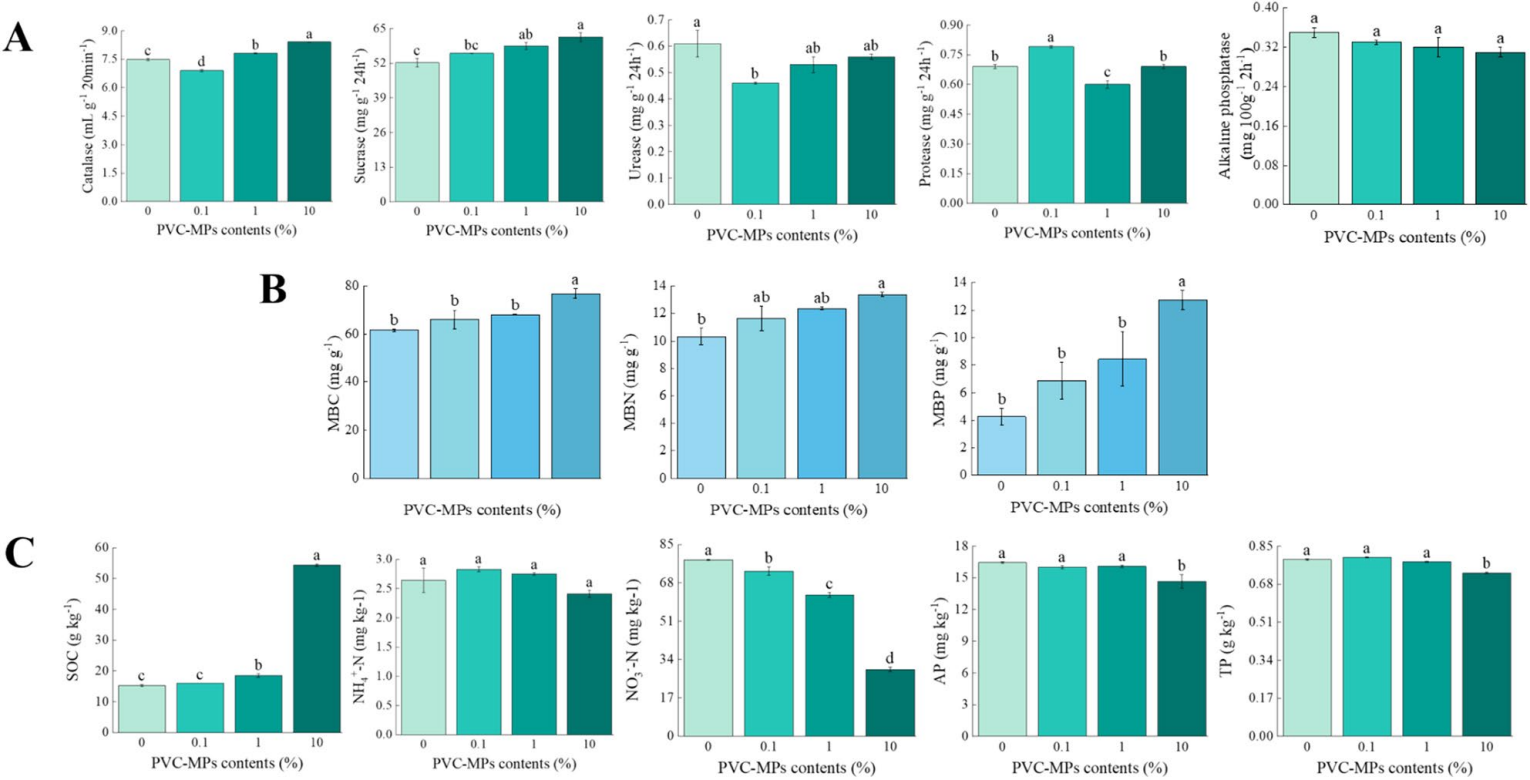
Effects of different PVC-MPs contents on soil enzymes (**A**), soil biomass (**B**), and soil nutrients (**C**) (MBC, MBC, and MBP represent soil microbial biomass carbon, nitrogen, and phosphorus, respectively; SOC, NH_4_^+^–N, NO_3_^−^–N, AP, and TP represent soil organic carbon, nitrate nitrogen, ammonium nitrogen, available phosphorus, total phosphorus, respectively; Different lowercase letters above histograms indicate statistical differences of soil enzymes, soil biomass, and soil nutrients among treatments at *P* < 0.05, respectively). One-Way ANOVA was used to analyze effects of PVC-MPs on soil properties and wheat growth and physiological parameters. They were presented in histogram. In each histogram, the different lowercases represent the significant difference at *P* < 0.05 level.

With the increase content of PVC-MPs, catalase and sucrase activities increased obviously (*P* < 0.01), while the activity of urease decreased significantly (*P* < 0.05) (Fig. 2A). Protease activity showed first increase and then decrease variation and the maximum value of protease activity occurred at 0.1% PVC-MPs (*P* < 0.01) (Fig. 2A). The activity of alkaline phosphatase showed a decrease trend, however, it has no significant difference among treatments (*P* > 0.05) (Fig. 2A). With the increase content of PVC-MPs, MBC, MBN and MBP increased significantly (*P* < 0.05) (Fig. 2B). Compared to the control, MBC, MBN, and MBP of the treatment at 10% PVC-MPs increased by 24.85%, 29.89% and 197.06%, respectively. With the increase of PVC-MPs, SOC increased significantly (*P* < 0.01) (Fig. 2C), The increase addition of PVC-MPs caused a significant decrease of soil NO_3_^−^–N (*P* < 0.01). Soil NH_4_^+^–N showed the first increase and then decrease trend (*P* > 0.05) (Fig. 2C). Similarly, the increase addition of PVC-MPs also resulted in a significant decrease of soil AP and TP (*P* < 0.05), however, soil AP and TP content have no significant difference among control, 0.1%, and 1% PVC-MPs treatments (Fig. 2C).

**Figure 3:**
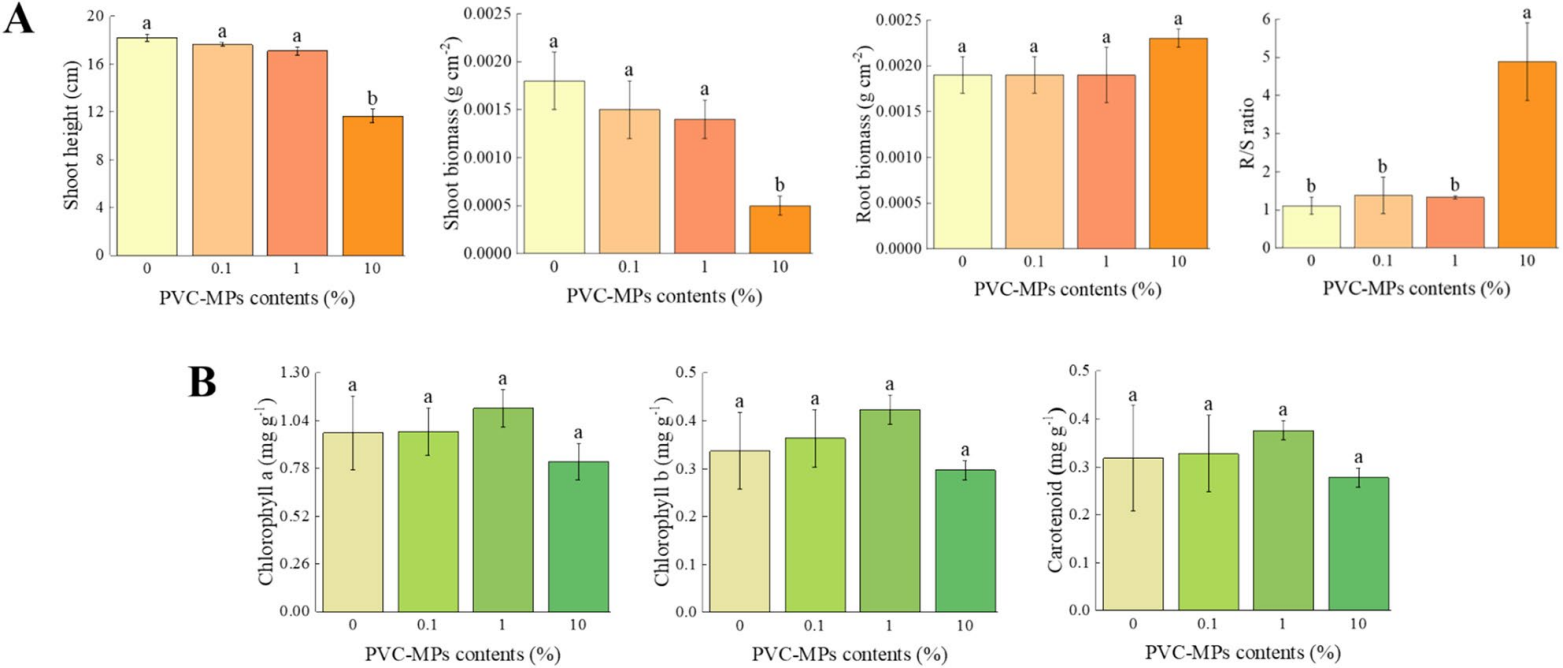
Effects of PVC-MPs on wheat seedlings growth traits and chlorophylls contents (R/S represents the ratio of root biomass to shoot biomass; Different lowercase letters above histograms indicate statistical differences of wheat seedlings growth traits and chlorophylls contents among treatments at *P* < 0.05, respectively). One-Way ANOVA was used to analyze effects of PVC-MPs on soil properties and wheat growth and physiological parameters. They were presented in histogram. In each histogram, the different lowercases represent the significant difference at *P* < 0.05 level.

**Figure 4:**
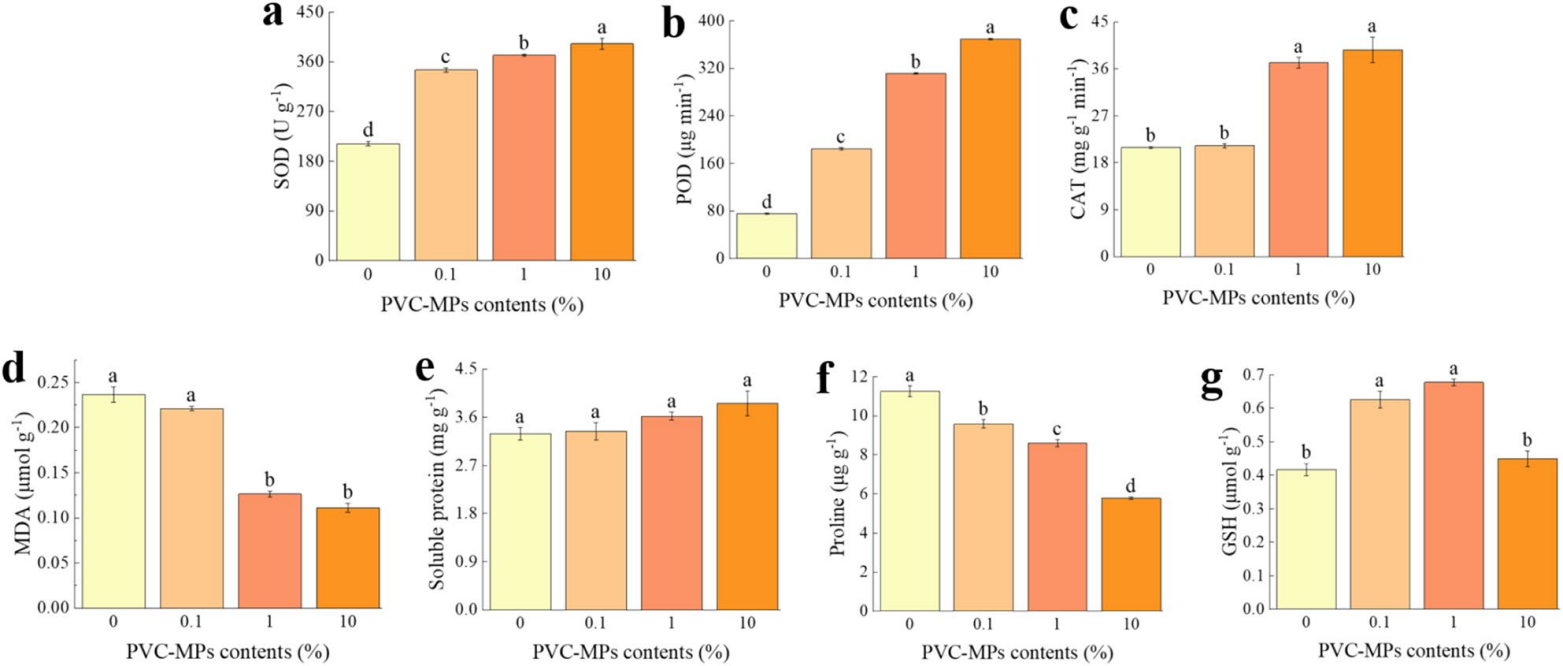
Effects of PVC-MPs on antioxidant enzymes and non-oxidants in wheat seedlings leaves (SOD, POD, CAT, MDA and GSH represent superoxide dismutase, peroxidase, catalase, malondialdehyde, and glutathione, respectively; Different lowercase letters above histograms indicate statistical differences of antioxidant enzymes and non-oxidants in wheat seedlings leaves among treatments at *P* < 0.05, respectively). One-Way ANOVA was used to analyze effects of PVC-MPs on soil properties and wheat growth and physiological parameters. They were presented in histogram. In each histogram, the different lowercases represent the significant difference at *P* < 0.05 level.

Compared to the control, 0.1% and 1% PVC-MPs had no significant influence on wheat seedlings shoot height (*P* > 0.05), while seedlings shoot height decreased significantly at 10% PVC-MPs treatment (*P* < 0.01) (Fig. 3A). With the increase of PVC-MPs, seedlings shoot biomass decreased and the significant difference appeared at 10% PVC-MPs treatment (*P* < 0.05). The seedlings root biomass has no significant variation among the treatments, even though it was slightly promoted at 10% PVC-MPs treatment than control (*P* > 0.05) (Fig. 3A). The R/S ratio was increased with increasing PVC-MPs and the increase degree of R/S ratio at 10% PVC-MPs reached more than four times than control (*P* < 0.05) (Fig. 3A). Chlorophyll *a*, chlorophyll *b*, and carotenoid contents in wheat seedlings leaves have no significant difference among the treatments (*P* > 0.05), even though these photosynthetic pigments showed the first increase and then decrease trend with the increase content of PVC-MPs (Fig. 3B). With the increase content of PVC-MPs, SOD, POD, and CAT activities in wheat seedlings leaves increased significantly (*P* < 0.05) (Fig. 4a–c), while MDA content decreased significantly (Fig. 4d). The content of soluble protein increased firstly and then decreased with the maximum value of soluble protein content occurred at 1% PVC-MPs (*P* < 0.05) (Fig. 4e). Proline content decreased obviously with the increase content of PVC-MPs (*P* < 0.05), while GSH content showed increase trend, even it has no significant difference the treatments (*P* > 0.05) (Fig. 4f,g).

**Figure 5:**
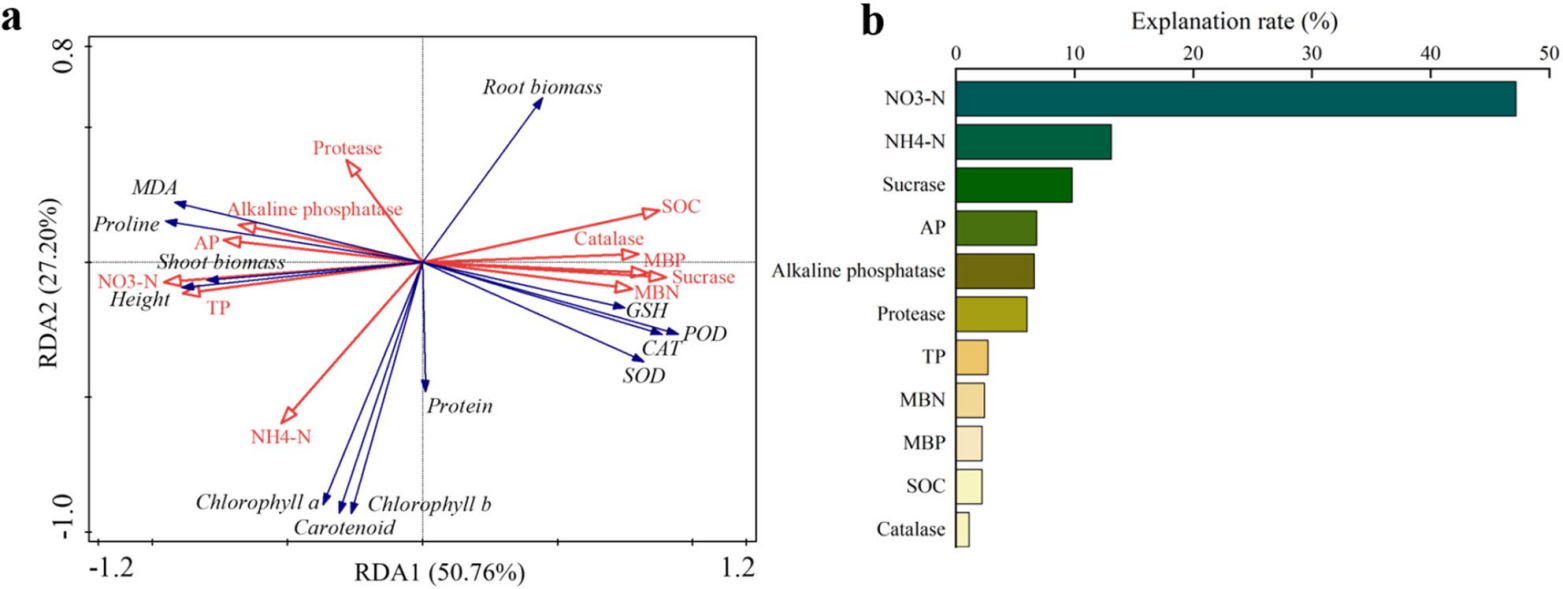
Redundancy analysis of soil properties on wheat growth and physiological parameters under the stress of PVC-MPs (SOD, POD, CAT, GSH, and MDA represent Superoxide dismutase, peroxidase, catalase, glutathione, and malondialdehyde, respectively; MBC, MBN, and MBP represent microbial biomass carbon, nitrogen, and phosphorus, respectively; SOC, NO_3_^−^–N, NH_4_^+^–N, AP, and TP represent soil organic carbon, nitrate nitrogen, ammonium nitrogen, available phosphorus, and total phosphorus, respectively.) One-Way ANOVA was used to analyze effects of PVC-MPs on soil properties and wheat growth and physiological parameters. They were presented in histogram. In each histogram, the different lowercases represent the significant difference at *P* < 0.05 level.

The effects of soil properties on wheat seedling traits by RDA analysis was displayed in Fig. 5 and Supplementary Table 2. The first axis explained 50.76% variation of wheat seedlings traits and the second axis explained 27.20% (Fig. 5a). Based on the simple term effects, NO_3_^−^–N (47.2%), TP (42%), sucrase (41.8%), SOC (41.6%), MBP (35.9%), catalase (34.4%), MBC (34.2%), MBN (32%), and AP (29.8%) had significant effects on wheat seedlings trait (*P* < 0.05) (Supplementary Table S2). Nevertheless, based on the conditional term effects, only soil NO_3_^−^–N had significant effect on wheat seedling traits (*P* < 0.05) with the highest explanation rate of 47.2% (Fig. 5b).

## Discussion

Crystalline can be sued to describe the structure of a polymer and the degree of crystallinity of plastic particles can be well-represented by XRD feature^14^. In general, plastic particles with high degree of crystallinity has sharp diffraction peak^22^. PVC-MPs in present study did not show apparent diffraction peak, indicating that PVC-MPs had lower degree of crystallinity and higher ability and rate of sorption^23^. However, the diffraction peak of all treatments occurred at 27° and the substance here was SiO_2_, based on the compare to Joint Committee on Powder Diffraction Standards cards for XRD analysis. Moreover, the degree of crystalline of soil was promoted by 0.1% and 1% PVC-MPs, which might result in the lower adsorption capacities of soil, and hence affecting microbial activities and soil properties^24^. The degree of crystalline of soil was inhibited by 10% PVC-MPs, which probably relates to the high content of PVC-MPs. The Fourier transform infrared spectroscopy is an important analytical method to identify the important functional groups associated with particles. In present study, compared to the control, no new function groups generated in soil under the stress of PVC-MPs, while the characteristic peaks were enhanced by 10% PVC-MPs. Hence, based on the XRD and FTIR analysis, we deduce that PVC-MPs will have more obvious effects on soil structure than on soil substance composition, which might be explained by the lower degradation of PVC-MPs due to the short period of the experiment.

Consequently, soil MBC, MBN, and MBP were promoted by PVC-MPs, which might be that the carbon-based composition of PVC-MPs can influence soil microbial processes, due to the fact that MPs are particles that contain about 80% carbon^25^. Under the stress of PVC-MPs, the increase of soil catalase activity can be used for protecting cell damage caused by hydrogen peroxide^11^. Meanwhile, catalase is an important intracellular enzymes found in aerobic organisms and the increase of soil catalase activity might relate to the increase of soil aerobic microbes, as it has been suggested that an increase in porosity by microplastics may increase the flow of air in soil, which will benefit for aerobic microbes growth^10^. The increase of sucrase activity suggests that PVC-MPs promoted the decomposition of soil organic matter, while the changes of urease, protease and alkaline phosphatase activities indicates that PVC-MPs inhibited the cycle of nitrogen and phosphorus^26^.

The increase of SOC content under the stress of PVC-MPs might be caused by the large amount of carbon-base in PVC-MPs^5^. Although the available nutrients in soil microbial biomass increased under the stress of PVC-MPs, soil available nitrogen and phosphorus among treatments have no significant variation. The possible explanation might be that PVC-MPs induced microbial immobilization of essential nutrients and soil microbes also need to absorb nutrients for their own growth^27^. The decrease of soil adsorption capacity caused by PVC-MPs could lead to the leaching of available nutrients, which is supported by the significant decrease of NO_3_^−^–N^28^. Additionally, the altered soil structure by PVC-MPs together with the alteration of soil microorganisms and soil enzymes will inhibit the nutrient cycling, which is consistent with the effect of polyethylene on soil available nitrogen^29^.

Seedlings growth is the most direct behavior for plant under the stress of microplastics^16^. PVC-MPs has been considered as the most toxic material on plants than other plastics that are frequently found in agricultural soil^13,30^. In present study, the lower content of PVC-MPs had no significant effect on the root biomass of wheat seedlings, which is similar to the weak effect of highly density polyethylene (2%) on shallot and the insignificant effect of polylactic acid (0.1% and 1%) on maize biomass^9,14^. The promotion effect of 10% PVC-MPs on wheat seedlings root biomass demonstrates that higher content of PVC-MPs still has no inhibitive effect, and conversely, it stimulated the wheat seedlings root biomass, which is consistent with the observations of maize seedlings that exposure to 10% polyethylene with the size of 100–154 μm^14^. However, compared to the root biomass, PVC-MPs had more severe effect on shoot biomass of wheat seedlings, which in line with the study on garden cress that exposure to polyethylene and the mixture of polyethylene and PVC^31^. Moreover, the weak effect of PVC-MPs on wheat seedlings root biomass and the strong inhibition of PVC-MPs on shoot biomass lead to a remarkable increase in the R/S ratio. It is noteworthy that the R/S ratio represented the allocation strategy for plants to optimize the resource use under a shortage of nutrient supply environment^32^. Meanwhile, the NO_3_^−^–N content in present study was significantly limited and the available phosphorus also was constricted to some extent. Therefore, we can infer that wheat seedlings are prone to ensure root growth under the limited available nutrients condition that caused by PVC-MPs.

Generally, the stable chlorophylls are essential for plant photosynthesis and the damage effects of abiotic stresses on the biosynthesis of chlorophylls have been confirmed^14^. In present study, PVC-MPs did not affect the chlorophylls of wheat seedlings significantly and it is in agreement with the effects of PVC-MPs and low density polyethylene on *Lepidium sativum* and wheat^31,33^. However, to some extent, compared to the control, the lower contents of PVC-MPs stimulated the chlorophylls, indicating the dose-dependent phenomenon of hormesis by PVC-MPs on chlorophylls and also suggesting that the growth of wheat seedlings is not coincide with the variation of photosynthetic parameters^34^. The significant increase of antioxidative enzymes, including SOD, POD and CAT, in present study indicates that wheat seedlings had a stronger antioxidant defense strategy in response to PVC-MPs stress and suggests the synergistic effect of those enzymes in protecting organelles and decreasing tissue damage^35,36^. Soluble protein is also a main osmotic regulator and GSH is an index to alleviate the toxic effects by abiotic stress^37^. In accordance with the variation of chlorophylls, soluble protein also increased at lower contents of PVC-MPs and decreased at high content of PVC-MPs and GSH content also increased to alleviate the stress of PVC-MPs. In general, as a major product of lipid peroxidation, MDA content reflects the degree of cell membrane damage that caused by oxidative stress^3^. However, in present study, MDA decreased with increasing PVC-MPs, suggesting that the lower oxidative damage to wheat seedlings leaves by PVC-MPs. The possible reasons for it are as follows: (1) Among enzymatic antioxidant processes, SOD is the first antioxidant defense against ROS and SOD increased significantly under PVC-MPs stress in present study^38^, (2) Chloroplasts are the primary photosynthetic organisms and also the major sites of ROS generation under environmental stress^39^. Previous studies displayed the reduced leaf chlorophyll levels that are caused by environmental stress, due to lipid peroxidation that causes oxidative damage to chloroplast organs^3,4^. In present study, chlorophylls in wheat seedlings leaves were not affected significantly by PVC-MPs, and even chlorophylls increased under the lower contents of PVC-MPs, which further suggests the lower oxidative damage by PVC-MPs. The accumulation of proline acts as an osmolyte for osmotic adjustment in plant under environmental stress^40^. The decrease of proline in present study also indicates wheat seedlings leaves is not severely impacted by PVC-MPs, which could be explained by the higher proline degradation in leaves or the wheat seeds in present study are sensitive to PVC-MPs^41^.

Nitrogen is one of the most critical limiting nutrient and the demand for nitrogen during plant growth, absorbed in the form of nitrate nitrogen or ammonium, is significantly higher than that of other elements^12,42^. The conditional effects of RDA analysis showed that soil NO_3_-N could explain 47.2% of the variation in growth and physiological parameters of wheat seedlings under PVC-MPs stress. Thus, it can be seen that the depletion of NO_3_^−^–N that caused by PVC-MPs is a key limiting factor for the wheat seedlings performance. The possible reason might be that soil porosity will increase with increasing content of PVC-MPs, which is usually attributed to leaching of nitrate nitrogen to the deeper soil^28^. Moreover, the variations of soil enzyme activities by PVC-MPs can reflect the microbial community and function under abiotic factors in soil to a certain extent^43,44^. Consequently, the alteration of microbial community might be potentially related to the changes of ammonium-oxidizing microorganisms and the copy number of *amo*A, which resulting in the dynamics of NO_3_^−^–N^45^. Therefore, to uncover the mechanism of PVC-MPs on wheat seedlings performance, it is critical to elucidate the changes in nitrogen processes caused by PVC-MPs in our future study.

## Conclusion

In present study, the ecological effects of PVC-MPs on soil properties and wheat seedling parameters were analyzed and the limiting factors on wheat seedling traits was discussed. Through the XRD and FTIR analysis, the new crystalline and functional groups did not appear in the soil under PVC-MPs stress, indicating that PVC-MPs have greater influence on soil structure than on soil substance composition. The changes in soil enzymes activity, soil microbial biomass, and soil nutrients under the stress of PVC-MPs were found. In order to defend against the effects of PVC-MPs, the antioxidant enzyme activities were improved. However, chlorophylls were affected by PVC-MPs, and MDA and proline decreased, indicating that the wheat seedlings are not severely impacted by PVC-MPs. More importantly, soil NO_3_^−^–N is a primary limiting factor for the performance of wheat seedlings under PVC-MPs stress. Overall, microplastics have negligible effects on soil–plant system. There is a dire need to establish long-term studies, such as the whole growth cycle for wheat, for a better understanding of the risk mechanisms of microplastics in realistic environment scenarios for safe agricultural functions.

### Supplementary Information

Supplementary Information.

## Data Availability

The original contributions presented in the study are included in the article, further inquiries can be directed to the corresponding authors.
