# Supplementary material for: Wheat (Triticum aestivum L.) seedlings performance mainly affected by soil nitrate nitrogen under the stress of polyvinyl chloride microplastics
Source: Sci Rep. 2024 Feb 29;14:4962. doi: 10.1038/s41598-024-54838-8 (PMC10904377; doi:10.1038/s41598-024-54838-8)
Supplement: Supplementary file 1 — Supplementary Information. [file 41598_2024_54838_MOESM1_ESM.pdf]

Title Page

Wheat (*Triticum aestivum* L.) seedlings performance mainly affected by soil nitrate nitrogen under the stress of polyvinyl chloride microplastics

Authors: Ke Zhang[a,b*], Yi Li[a], Mengge Wang[a], Xu Zhang[a], Kangqinglin Xiao[a], Chuang Ma[a,b], Xiaojing Zhang [a,b], Hongzhong Zhang[a,b], Yongle Chen[c*]

[a]*School of Material and Chemical Engineering, Zhengzhou University of Light Industry, Zhengzhou 430000, China*

[b]*Collaborative Innovation Center of Environmental Pollution Control and Ecological Restoration, Henan Province, Zhengzhou 430000, China*

[c]*College of Earth and Environmental Sciences, Lanzhou University, Lanzhou 730000, China*

Supplementary Tables

Table 1 Properties of the test soil (EC, SOC, TN, AP, and TP represent electrical conductivity, soil organic carbon, total nitrogen, available phosphorus, and total phosphorus)

pH	EC (μs cm^{-1})	SOC (g kg^{-1})	TN (g kg^{-1})	AP (mg kg^{-1})	TP (g kg^{-1})
7.34	184	20.04	0.71	19.48	0.822

Table 2 Simple term effects and conditional term effects of Redudancy anlaysis (MBC, MBN, and MBP represent microbial biomass carbon, nitrogen, and phosphorus, respectively; SOC, NO_3^--N, NH_4^+-N, AP, and TP represent soil organic carbon, nitrate nitrogen, ammonium nitrogen, available phosphorus, and total phosphorus, respectively.)

Simple Term Effects:			
Name	Explains (%)	pseudo-F	*P*
NO_3^--N	47.2	8.9	0.026
TP	42	7.2	0.026
Sucrase	41.8	7.2	0.026
SOC	41.6	7.1	0.026
MBP	35.9	5.6	0.032
Catalase	34.4	5.3	0.032
MBC	34.2	5.2	0.036
MBN	32	4.7	0.04
AP	29.8	4.3	0.026
Alkaline phosphatase	25.6	3.4	0.112
NH_4^+-N	24.1	3.2	0.112
Protease	10.4	1.2	0.624
Urease	6.4	0.7	0.624

Supplementary Figures

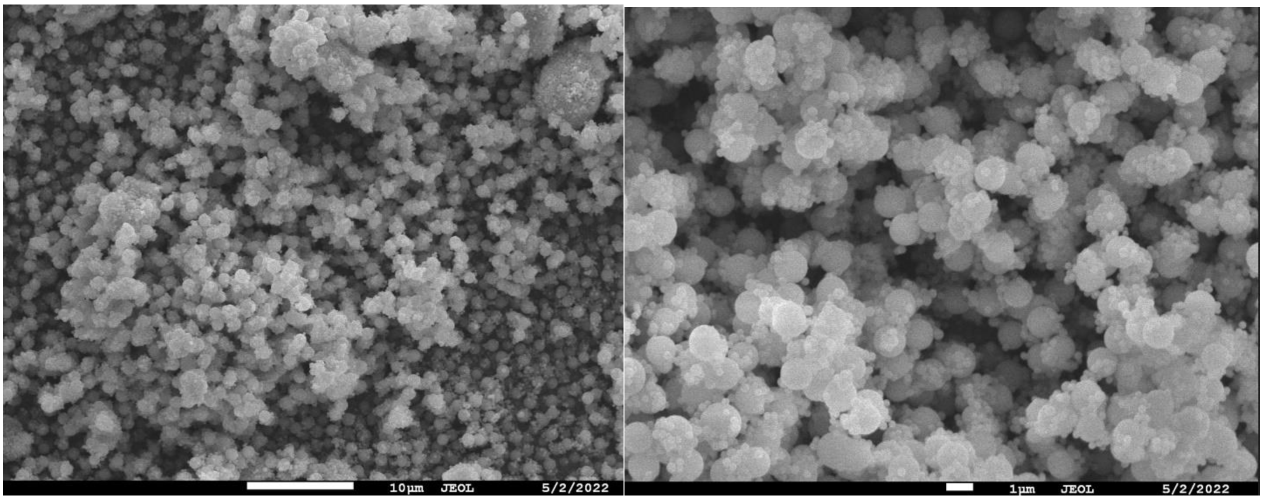

Fig. 1 The morphology of PVC-MPs is characterized by scanning electron microscopy
